# To Dr. Kinglake

**Published:** 1803-02-01

**Authors:** 


"To Dr. KINGLAKE.

"Sir,

"I was exceedingly gratified on reading your very ingenious paper on the cure of arthritis, inserted in the last Number of the Physical Journal,* particularly as it contained the same sentiments on the subject, as I have long entertained in my own mind. I confess I was a long time shackled with *old* opinions, and found it very difficult to adopt the noble declaration of Horace,

'Nullius addictus jurare in verba majestri.'

Still more to venture on the trial of a new mode of treating gout. However, at length I made the experiment *last August;* when being called to a Mr. H. who had resided many years in Jamaica, I found him attacked with gout for the second time in both great toes. The redness and pain were great, and the skin was smooth and shining. I directed a strong solution of sal. ammon. c. to be constantly applied with folds of linen to the affected parts, kept his body open with kali tartaris, and occasionally gave an opiate. He pursued this plan, and was quite well at the end of a fortnight. This is the only case in which I have made trial of the plan; but if in any future communications to the public, you should think this single testimony will corroborate your own evidence on the subject, you are at liberty to make what use you please of it.

"I have the honour to be, &c.

"G. CUSTANCE, Surgeon."

"*Kidderminster, Dec.* 5, 1801."

---

"Sir,

"I duly received your favour of the 26th of December, 1801; and, in compliance with your request, I have the pleasure to acquaint you, that I have lately been successful in three cases of rheumatic fever, by the application of cold to the parts that were swelled and inflamed.

"The form I used was ℥ss of sal. ammon. c. dissolved in aquæ ℔j. Rags well soaked in this solution were kept constantly to the parts, and the patients expressed their surprise at the ease they experienced from the application, and the good effects were soon visible in reducing the swelling

---

* Numb. XXXIII. p. 434.

swelling and inflammation. I am now so fully convinced of the perfect safety and utility of the application of cold to rheumatic and arthritic inflammation, that I shall never in future hesitate to recommend it. I had intended communicating my thoughts on the subject to the Editors of the Medical and Physical Journal; but supposing you have it in contemplation to favour the public, in some shape or other, with your own observations, I prefer giving a statement of simple facts to you, which you are welcome to make what use of you judge proper.

" I am, &c.

" *Kidderminster, April* 15, 1802." " G. CUSTANCE."

---

An Address, by Dr. Kinglake.

Dr. Kinglake presumes, from the *original view* which he has submitted to the public, in his two papers *on the Nature and Cure of Gout,** to request *generally* his Medical Brethren, to communicate to him any intelligence which correct experience might furnish, of the effects of his new mode of treatment, that he might be enabled, at no distant period, to present a mass of evidence, competent to determine, *beyond all doubt*, whether the arthritic patient must continue to be doomed to languish under the lingering and indefinite torture of an uncontrollable malady, or safely avail himself of a prompt and efficacious remedy.

The conjoint aid of the medical faculty in this investigation, is almost indispensably necessary, to countervail the insurmountable difficulties which would be opposed to a solitary endeavour, by the inveteracy of popular prejudice, against the employ of *topical cold*, in an affection which has hitherto been supposed peculiarly to require an unremitted increase of both *local heat* and *systematic excitement*.

*Taunton, Dec.* 22, 1802.

---

* See Med. and Phys. Journal, Numb. XXXIII. p. 434.—Numb. XLVII.

*Observations*

